# Matrix effect of four Chinese medicinal herbs on colloidal gold immunoassay for organophosphorus pesticides

**DOI:** 10.1515/biol-2025-1201

**Published:** 2026-02-12

**Authors:** Maoqiong Wei, Ziri Wang, Li Wang, Shanshan Lan, Zhenhuan Liu, Xin Lin, Hongcheng Liu

**Affiliations:** Quality Standards and Testing Technology Research Institute, Yunnan Academy of Agricultural Sciences, Kunming 650205, China; School of Medicine, Yunnan University of Business Management, Kunming, 650106, China

**Keywords:** gold immunochromatographic assay, Chinese medicinal herb, matrix effect, organophosphorus pesticides

## Abstract

The matrix effect (ME) presents a serious problem for the immunoassay of pesticide residues, considerably limiting its practical application in Chinese medicinal herbs and other food products. The objective of this study was to achieve reduced matrix effect and improved detection accuracy of colloidal gold test strips. For that reason, four types of Chinese medicinal herbs (*Ophiopogon japonicus*, *Bulbus Fritillaria*, *Polygonatum sibiricum,* and *Folium Artemisiae Argyi*) were used as samples to investigate the effect of the Chinese medicinal herb matrix on the gold immunochromatographic assay (GICA) for analyzing three organophosphorus pesticide (chlorpyrifos, triazophos, and isocarbophos) residues. In the quantitative analyses of chlorpyrifos, triazophos and isoprocarbophos, the extraction method completely eliminated the matrix interference, and the sensitivities of GICA were increased by about 8.3, 1.7 and 1 times, respectively, compared with those of the standard operating instructions for test strips. The mechanism of the matrix of Chinese herbal medicines on the ME of colloidal gold test strips was preliminarily explored. It is reasonable to hypothesize that natural products and secondary metabolites have a significant effect on GICA, leading to ME.

## 1 Introduction

Traditional Chinese medicinal herbs are mainly grown artificially in mountainous and forested regions, where pest infestations are common. To ensure the yield of these herbs, farmers often turn to using pesticides for obtain high yield. However, excessive pesticide usage can result in pesticide residues. Furthermore, pesticides are extensively employed during the cultivation of crops such as wheat, rice, fruits, and vegetables [1]–3]. With continuous use of various pesticides, the accumulation of pesticide residues in the soil increases. Three organophosphorus (OP) pesticides – chlorpyrifos, triazophos, and isocarbophos – are used to kill insects in plants. In our previous study, these three pesticides were detected with high frequency, therefore, this experiment took these three pesticides as the object of study. These pesticides can cause neurological dysfunction by inhibiting the activity of acetylcholinesterase, thus achieving insecticidal efficacy. However, continuous use of these OP pesticides has caused numerous issues in biological systems and the environment [4]–6]. Therefore, precise detection of chlorpyrifos, triazophos, and isocarbophos pesticide residues in foods is of great significance for ensuring food safety and promoting people’s health. Conventional methods for detecting pesticides to ensure food safety include gas chromatography, high-performance liquid chromatography, liquid chromatography–tandem mass spectrometry (LC–MS/MS), enzyme inhibition methods, and enzyme-linked immunosorbent assays (ELISAs) [7]. The key component of a biosensor is its recognition element, Although the instrumental methods offer high sensitivity, they involve high detection costs and long detection times and require expensive equipment and professional personnel to operate, which are not conducive toon-site detection [8]. Despite their fast detection speed and straightforward operation, both the enzyme inhibition methods and ELISA exhibit low sensitivity and are prone to providing false negative results [9]. Therefore, it is crucial to develop a straightforward and effective method for detecting pesticides with a short detection time, low cost, and suitability for on-site testing. One such method is gold immunochromatographic assay (GICA) that provide a rapid and accurate means for assessing the presence of harmful substances, enabling timely intervention measures [10]. The key component of a GICA is its recognition element, antibodies are one of the recognition elements which have been preferred choices of researchers for a long time due to their high specificity, affinity and versatility [11], 12]. Chinese medicinal herbs absorb and accumulate pesticides from residues in soil and irrigation water, resulting in pesticide levels exceeding regulatory limits [13], 14]. *Polygonatum sibiricum Delar. ex Redoute*, which belongs to the family Asparagaceae [15], is known for itsnumerous biological activities, such as antioxidant activity and anti-aging activity, attributed to the presence of various components, including alkaloids, flavones, steroid saponins, lignins, aminoacids, and *Polygonatum sibiricum* polysaccharides (PSP) [16], 17]. During the cultivation of *P. sibiricum*, pesticides are unavoidably used to combat pests and diseases, leading to issues of pesticide residues. This poses health risks to consumers and hampers the sustainable development of the *P. sibiricum* industry [1], 16]. *Ophiopogon japonicus* (L.f) Ker-Gawl, =, also belongs to the family Asparagaceae [18], 19]. It is cultivated for its dried tuberous roots and may be susceptible to diseases such as root rot and powdery mildew, necessitating the use of pesticides such as chlorpyrifos for prevention of such diseases. *Fritillaria ussuriensis Maxim.* from the genus Fritillaria in the family Liliaceae, is rich in alkaloids, saponins, flavonoids, and other secondary metabolites [20]. *Folium Artemisiae Argyi*, from the genus Artemisia in the family Asteraceae, is the dried leaves of the Artemisia plant and isa traditional Chinese medicinal herb. These leaves contain active compounds such as flavonoids, phenolic acids, and terpenes [21].

The study identified high-frequency restricted pesticides in the Chinese medicinal herbs *P. sibiricum*, *O. japonicus*, *F. ussuriensis Maxim*, and *F. Artemisiae Argyi*: chlorpyrifos, isocarbophos, and triazophos. The issue of pesticide residues in Chinese medicinal herbs is notably intricate, highlighting the urgent need to establish detection methods using colloidal gold and preprocessing techniques for prohibited and restricted pesticides in medicinal herbs. This endeavor is crucial for ensuring quality and safety standards in Chinese medicinal herbs.

Pesticide residue analysis is part of trace analysis, and the secondary metabolites found in Chinese medicinal herbs introduce substantial matrix interferences during pesticide residue analysis. Therefore, selecting appropriate preprocessing method for complex matrices becomes crucial to improve the extraction efficiency of target pesticides in medicinal herbs, while minimizing the impact of matrix effects (MEs) on antigen–antibody binding interactions [22]. Addressing these technical challenges is imperative for pesticide residue detection in Chinese medicinal herbs.

The interference known as the “matrix effect” induced by complex components in the matrix significantly disrupts immune responses, impacting the sensitivity, accuracy, and efficiency of analysis. This interference poses a serious challenge to the precise quantification of target analytes [23]. In the process of pesticide residue detection, a single pesticide can exhibit diverse MEs across different types of Chinese medicinal herbs and different pesticides can result in varying MEs within the same matrix. Currently, research on organophosphate (OP) MEs predominantly focuses on instrumental aspects, particularly in relation to vegetable matrices. Studies addressing the effects of Chinese medicinal herb matrices on GICA are limited. Therefore, four Chinese herbal medicines were selected in this study to evaluate the MEs of three OP pesticides in different matrices using GICA, which can help to reduce the false-positive and false-negative rates of detection of Chinese herbal medicines in GICA. The aim of this study is to provide valuable insights into the detection of pesticide residues in herbal medicines and ultimately improve the accuracy of such analyses.

## 2 Materials and methods

### 2.1 Chemical reagents and materials

Guaranteed reagent (GR) Acetonitrile, n-hexane, and acetone were purchased from Merck KGaA, Germany (Darmstadt, Germany). Distilled water was prepared using an GenPure Pro UV/UF ultrapure water system (Waltham, MA, USA). Analytical grade sodium chloride and magnesium sulfate were obtained from Tianjin Fengchuan Reagent Technology Co., Ltd (Tianjin, China). The kits for GICA for analyzing chlorpyrifos, triazophos, and isocarbophos were sourced from Anxinbao Company (Shenzhen, China). The selected medicinal herbs, namely, *P. sibiricum*, *O. japonicus*, *F. ussuriensis*, and *F. Artemisiae Argyi*, were procured from Fujun Chinese Medicinal Herbs Store in Kunming, Yunnan Province, China. The pesticide standards of chlorpyrifos, Isocarbophos and triazophos were purchased from Tianjin Alta Technology Co. Pesticide concentrations were all 1 mg/ml.

### 2.2 Major instruments and equipment

The N-EVAP112 nitrogen evaporator was imported from Organomation, USA (Kansas, USA). The digital ultrasonic cleaner KH-250DE was purchased from Kunshan Hechuang Ultrasonic Equipment Co., Ltd (Kunshan, China). The electronic balance BSA124S was obtained from Sartorius, Beijing, China). The high-speed centrifuge TGL-10B was acquired from Shanghai Anting Scientific Instrument Factory (Shanghai, China). The shaker (incubator) HY-5A was sourced from Jiangsu Kexi Instrument Co., Ltd (Changzhou, Jiangsu, China). The closed electric furnace DDF-1KW was purchased from Changzhou Jintan Liangyou Instrument Co., Ltd (Jiangsu, China). The vortex oscillator QL-861 was obtained from Thermo Scientific, Waltham, MA, USA.

### 2.3 Experimental conditions

#### 2.3.1 Preparation of control with standards

The pesticide standards of chlorpyrifos, Isocarbophos and triazophos were aspirated at 100 μg/ml, and the solution was fixed with methanol to form a stock solution of 40 μg/ml. From the stock solution, an intermediate solution with a concentration of 500 ng/ml was prepared. Subsequently, a series of pesticides solutions were prepared by spiking the pesticides standard solution in specialized diluent at different concentrations from the intermediate solution. The concentrations of the prepared control with standards are presented in Table 1.

**Table 1:** Concentrations of control with standards prepared.

OP pesticides	Concentrations prepared (ng/ml)			
Chlorpyrifos	25	50	100	250
Isocarbophos	10	20	100	200
Triazophos	50	100	250	500

#### 2.3.2 Solvent selection for extraction

For highly polar OP pesticides, it is recommended to use solvents such as dichloromethane, ethyl acetate, and acetone, which exhibit significant polarity. Acetonitrile is suitable for analyzing multiple pesticide residues. Therefore, this study focuses on two solvents: acetonitrile and amixture of n-hexane andacetone.

#### 2.3.3 Pretreatment methods

The matrix of traditional Chinese medicinal herbs is exceptionally complex, which greatly increases the challenges in detecting pesticide residues. Improper pretreatment can lead to significant MEs during analysis, thus impacting the accuracy of qualitative and quantitative pesticide analysis. Common pretreatment methods for testing pesticide residues in samples include sulfonation, solid-phase extraction (SPE), and gel permeation chromatography (GPC) [24]. In the 2020 edition of the *Chinese Pharmacopoeia,* direct extraction, the QuEChERS method, and SPE are mainly used to remove interfering substances such as fatty acids, pigments, and essential oils in pesticide detection for traditional Chinese medicine. However, preliminary experiments have shown that these methods do not provide satisfactory purification effects. Furthermore, the pretreatment process is complex and requires different handling methods for various types of pesticides.

##### 2.3.3.1 Test strip standard practice instruction method (method A)

1.0 g of powdered medicinal herb was placed in a 50-ml graduated centrifuge tube. Then, 3 ml of a specialized diluent were added, and the tube was tightly capped. The tube was manually inverted 60 times per minute to ensure thorough mixing. Subsequently, a 2-min sample wash was performed, followed by a 1-min settling period. The resulting mixture in the tube is referred to as the test solution. If further dilution was required, the sample solution and diluent were mixed in different proportions in a 5-ml centrifuge tube before testing to obtain the test solution (sample solution + diluent). Please note that different samples exhibit varying detection limits and dilution ratios.

##### 2.3.3.2 Acetonitrile heating method (method B)

1.0 g of powdered sample (passed through a No. 2 sieve) was weighed and placed into a 50-ml centrifuge tube, and then acetonitrile was added to make up the total volume of 20 ml. After vortex shaking with 1 g of NaCl for 1 h, 10 ml of the supernatant was transferred to a 50-ml beaker. This liquid was evaporated using a furnace set to setting 1 (about 70 °C), until the liquid is almost evaporated. After return to room temperature (20–30 °C), 3 ml of the specialized diluent was added to the bottom. Subsequently, detection of the homogenized liquids was carried out according to the method A.

##### 2.3.3.3 n-Hexane: acetone (1:1) nitrogen blowing method (method C)

1.0 g of the sample (passed through a No. 2 sieve) was weighed and then n-hexane: acetone (1:1) solution was added to make the volume up to 20 ml. After soaking for 15 min, the mixture was vortexed for 1 min and ultrasonicated for 30 min. Then, 4.0 g of MgSO_4_ and 1.0 g of NaCl were added and mixed thoroughly. The mixture was again vortexed and centrifuged for 3 min (4,000 rpm) to obtain a 3-ml supernatant, which was then subjected to nitrogen evaporation to near dryness in a 10-ml test tube. After adding 3 ml of specialized diluent and thoroughly mixing via vortexing, the test solution was appropriately diluted and tested according to the method A.

#### 2.3.4 Detection steps

The GICA and the sample solution for testing were allowed to return to room temperature (20–30 °C) before use (Remarks: Please keep the remaining microtiter wells sealed).

(1) Take 100 μl of the sample solution to be tested and drop it vertically into the microtiter wells, aspirate 4–5 times and incubate for 2 min;
(2) Take out the test card from the original package, open it and put it flat on the table, please use it within 1 h after opening;
(3) Aspirate all the liquid in the microwells, blow gently for 10 s, and then add all drops into the spiking hole of the test card;
(4) Start timing after adding the sample, read the result in 5∼8 min, other time reading is invalid.

#### 2.3.5 Actual sample spiking recovery testing

##### 2.3.5.1 Selection of extraction methods for chlorpyrifos from four Chinese medicinal herbs

(1) Spiking determination using method A

Control with standard testing was conducted according to Section 2.3.1, and the concentration of the spiked samples was determined based on the detection limit of the control with standard. The extraction method specified in Section 2.3.3.1 was used. Based on the detection limit of the control with standards, different Chinese medicinal herb matrices were subjected to the addition process which addition different concentrations of pesticides (Table 1), and the MEs on pesticide detection were analyzed. A preliminary experiment was conducted using *Polygonatum sibiricum*as the matrix, with the chlorpyrifos standard solution spiked at concentrations ranging from 20 to 300 ng/ml (Table 2). Once the appropriate spiking range was determined, spiking experiments were performed using *O. japonicus*, *Bulbus Fritillaria*, and *Folium Artemisiae Argyi* as matrices. The impact of MEs on the detection limit of chlorpyrifos was assessed based on the test results.

(2) Spiking determination using method C

**Table 2:** Design of GICA for control with standards detection.

Names of Chinese medicinal herbs	The spiking concentration of chlorpyrifos and the blank CK Unit (ng/ml)						
	300	250	200	50	30	20	CK
*Polygonatum sibiricum*	300	250	200	50	30	20	0
*Ophiopogon japonicus*	300	250	200	50	30	20	0
*Bulbus Fritillaria*	300	250	200	50	30	20	0

*Folium Artemisiae Argyi* contained impurities such as volatile oils, flavonoids, chlorogenic acid, and acidic polysaccharides that could not be detected using methods A and B. The eluent used in method C (hexane: acetone = 1:1) possessed strong ion-exchange capabilities, allowing it to remove fatty acids, organic acids, polar pigments, and metal ions. The recovery rate stabilized when the volume of hexane: acetone (1:1) exceeded 10 ml. The extract was a mixed organic solvent with good elution effect, and considering economic, environmental, and experimental requirements [24], n-hexane: acetone (volume ratio 1:1) was selected as the elution solvent for extraction, with a volume of 20 ml. The extraction method was as specified in Section 2.3.3.3. The extract was diluted according to Table 3, and the judgment of negative or positive was based on the test paper results.

**Table 3:** Design of method C for extracting chlorpyrifos (0 and 30 ng/ml) with different dilution factors from *Folium Artemisiae Argyi*

Name of Chinese medicinal herb	Dilution factor	
*Folium Artemisiae Argyi*	1:0	1:1

##### 2.3.5.2 Selection of extraction methods for triazophos from four Chinese medicinal herbs

(1) Spiking determination using method B

The extraction results when using method A revealed false negatives when positive test strips were spiked. Method B was then used for extraction. Control target testing was conducted according to Section 2.3.1 using the control target detection limit to determine the spiking concentration in actual samples. The extraction method mentioned in Section 2.3.3.2 was employed. Considering a control target detection limit of 100 ng/ml, concentrations of 100 and 200 ng/ml of triazophos were added to *Polygonatum sibiricum*, *O. japonicus*, and *Bulbus Fritillaria* Chinese medicinal herbs (Table 4). The extraction solution was tested without dilution. The impact of MEs on the detection limit of triazophos was assessed based on the test results.

(2) Spiking determination using method C

**Table 4:** Design of method B for extraction of triazophos from different Chinese herbal medicines.

Name of Chinese medicinal herbs	Prepared concentrations of triazophos (ng/ml)	
*Polygonatum sibiricum*	100	200
*Ophiopogon japonicus*	100	200
*Bulbus Fritillaria*	100	200
*Folium Artemisiae Argyi*	100	200

As method B proved in effective in detecting triazophos pesticide in *Folium Artemisiae Argyi* at a concentration of 100 ng/ml, method C was employed for extraction. The extraction solution was tested at dilutions of zero fold (Undiluted) and one fold, and compared with blank samples (Table 5).

**Table 5:** Design of method C for extraction of triazophos from *Folium Artemisiae Argyi*

Name of Chinese medicinal herb	Triazophos concentration ng/ml	Dilution factor	
*Folium Artemisiae Argyi*	100	1:0	1:1
	0	1:0	1:1

##### 2.3.5.3 Selection of extraction methods for isocarbophos from four Chinese medicinal herbs

(1) Spiking determination using method A

Control target testing was conducted in according to Section 2.3.1 to determine the spiking concentration in actual samples based on the control target detection limit. The extraction method specified in Section 2.3.3.1 was employed. The impact of MEs was assessed by analyzing the spiking concentration of the matrix based on the control target detection limit. Isocarbophos was added at concentrations of 20–50 ng/ml in *Polygonatum sibiricum*, *O. japonicus*, *Bulbus Fritillaria*, and *Folium Artemisiae Argyi* (Table 6).

(2) Determination of optimal extraction dilution factor for method A

**Table 6:** Design of method A for extraction of isocarbophos from different Chinese herbal medicines.

Name of Chinese medicinal herbs	Concentrations of isocarbophos ng/ml		
*Polygonatum sibiricum*	50	30	20
*Ophiopogon japonicus*	50	30	20
*Bulbus Fritillaria*	50	30	20
*Folium Artemisiae Argyi*	50	30	20

Based on the detection described in Section 2.3.3.1, false positives were observed during the blank detection of *Folium Artemisiae Argyi*. The experiment included the dilution of blank and spiking extraction liquids, with the dilution factors specified in Table 7.

**Table 7:** Design of method A for extraction of isocarbophos (0 and 30 ng/ml) with different dilution factors from *Folium Artemisiae Argyi*

Name of Chinese medicinal herb	Dilution factor		
*Folium Artemisiae Argyi*	1:1	1:2	1:5

#### 2.3.6 Calculation of matrix effects

Four samples of pesticide-free Chinese medicinal herbs were chosen to create matrix solutions using three pretreatment methods A, B, and C. These matrix solutions were then diluted to serve as different experimental treatments, known as matrix standards. A control sample, referred to as reagent standard, was created by diluting a reference standard sample with the diluent from method A. The lowest detection limit of the matrix standard, which was determined by comparing it to the control target concentrations of various pesticides, was considered as the matrix standard concentration. This matrix standard concentration was used to calculate the MEs for each sample. Matrix standard concentration refers to the lowest limit of detection of pesticides in Chinese herbal medicines by colloidal gold test strips. The formula used to calculate the MEs was as follows: ME (%)=(matrix standard concentration/control target concentration −1)×100. A weak ME was indicated when the ME fell within −20 % to 20 %, a moderate ME was observed when the ME ranged from −50 to −20 % or 20–50 %, and a strong ME occurred when the ME was either less than −50 % or greater than 50 % [1], 25].

## 3 Results

### 3.1 Determination of control with standards

The control with standards for chlorpyrifos ranged from 25 to 250 ng/ml, for triazophos from 10 to 200 ng/ml, and for isocarbophos from 50 to 500 ng/ml (Table 8). The detection limits of chlorpyrifos, triazophos, and isocarbophos in the control with standards were found to be 250, 100, and 50 ng/ml, respectively.

**Table 8:** GICA results for control with standards at different concentrations.

	Chlorpyrifos				Triazophos				Isocarbophos			
Concentrations (ng/ml)	25	50	100	250	10	20	100	200	50	100	250	500
Results	−	−	−	+	−	−	+	+	+	+	+	+
Control with standard minimum detection limit (ng/ml)	250				100				50			

negative (−), positive (+), not detected (ND).

### 3.2 Detection results of pesticides in four Chinese medicinal herbs

#### 3.2.1 Detection results of chlorpyrifos in four Chinese medicinal herbs

##### 3.2.1.1 Spiking test using method A

**Figure 1:**
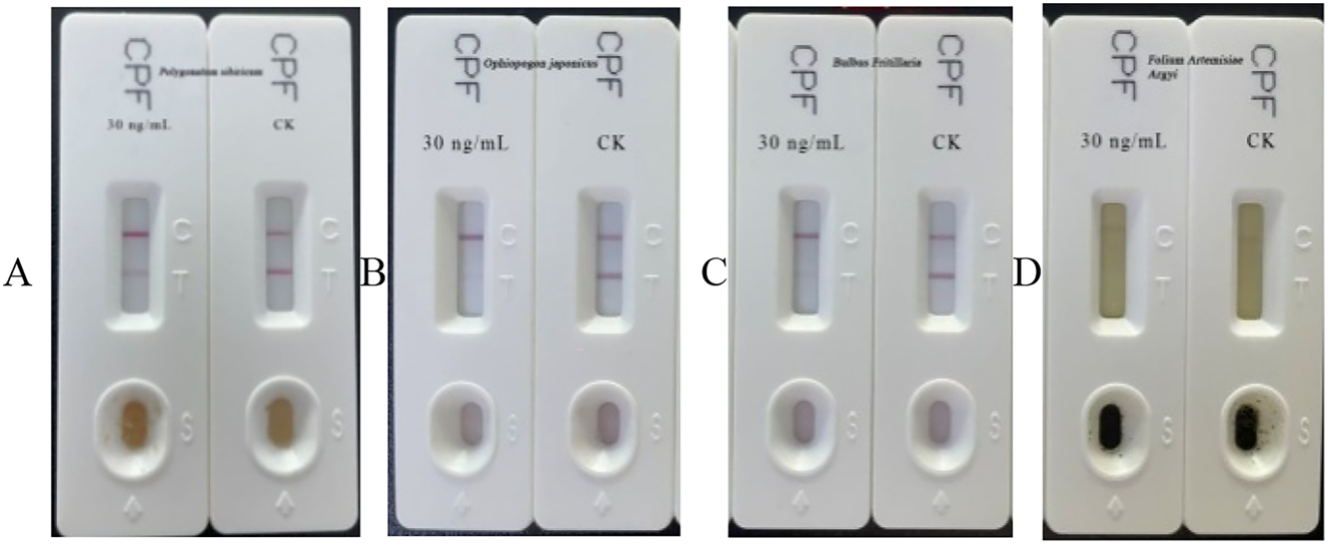
GICA results of chlorpyrifos at 30 ng/ml and 0 ng/ml (CK) in four Chinese medicinal herbs using method A. A: The addition of chlorpyrifos at concentrations of 30 ng/ml and CK to *Polygonatum sibiricum*. B: The addition of chlorpyrifos to ophiopogon japonicas at concentrations of 30 ng/ml and CK. C: The addition of chlorpyrifos to *Bulbus Fritillaria* at concentrations of 30 ng/ml and CK. D: The addition of chlorpyrifos to *Foliu* *m Artemisiae Argyi* at concentrations of 30 ng/ml and CK.

Incorporating a standard solution of three pesticides with concentrations ranging from 20 to 300 ng/ml into the four Chinese medicinal herbs, the specific concentrations added and results are detailed in Table 9. To investigate the potential impact of MEs on the sensitivity of test strips, an initial spiking experiment was conducted using pesticide concentrations exceeding 300 ng/ml. Results presented in Figure 1 consistently show detectable levels of 30 ng/ml, confirming that the concentration exceeded the detection limit of the test strips and that the MEs did not influence the experimental outcomes. Subsequent reductions in concentration to 250–20 ng/ml were performed. At a concentration of 20 ng/ml, all spiking test results yielded negative outcomes. However, at a concentration of 30 ng/ml, *Polygonatum sibiricum*, *O. japonicus*, and *Bulbus Fritillaria* exhibited negative controls and the spiking test results confirmed positive values for these samples (Figure 1A–C). Blank *Folium Artemisiae Argyi* and spiked *Folium Artemisiae Argyi* were tested when pesticide was extracted from *Folium Artemisiae Argyi* using method A at a spiking concentration of 30 ng/ml. Results indicate that the control sample was positive (Figure 1D). Upon dilution factors of 1:1 and 1:2, both spiking and blank tests were positive at 1:1 and negative at 1:2. Method A proved successful in extracting chlorpyrifos from *Polygonatum sibiricum*, *O. japonicus*, and *Bulbus Fritillaria* but was ineffective in accurately extracting chlorpyrifos from *Folium Artemisiae Argyi*. To mitigate false positives, a transition to method B for the extraction chlorpyrifos from *Folium Artemisiae Argyi* spiked with concentrations of 30 and 50 ng/ml was undertaken. After a two fold dilution of the extraction solution, *Folium Artemisiae Argyi* still exhibited false positives in the control sample. Further increasing the dilution factor led to negative results in the control and the spiked samples, indicating that method B was unsuitable for the pretreatment extraction chlorpyrifos from *Folium Artemisiae Argyi*.

**Table 9:** GICA results for spiking test at different concentrations in four Chinese medicinal herbs.

Name of Chinese medicinal herbs	Spiking concentration and blank comparative CK of chlorpyrifos (ng/ml)						
	300	250	200	50	30	20	CK
*Polygonatum sibiricum*	+	+	+	+	+	−	−
*Ophiopogon japonicus*	+	+	+	+	+	−	−
*Bulbus Fritillaria*	+	+	+	+	+	−	−
*Folium Artemisiae Argyi*	+	+	+	+	+	+	+

negative (−), positive (+), not detected (ND).

##### 3.2.1.2 Spiking test using method C

**Figure 2:**
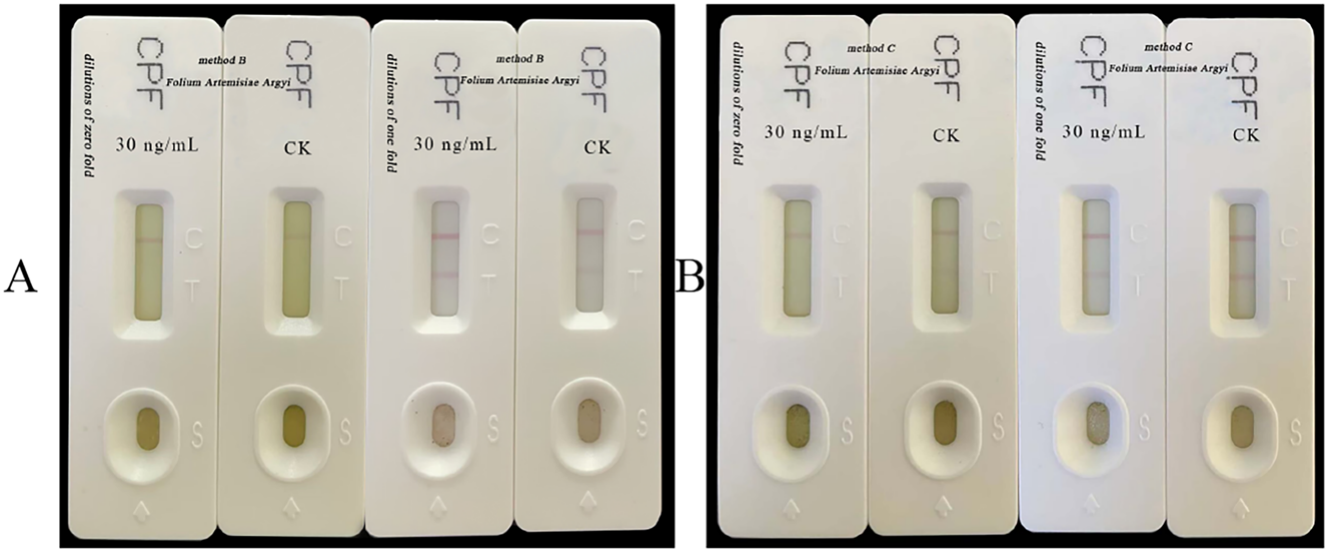
GICA results of chlorpyrifos at 30 ng/ml in *Folium Artemisiae Argyi* using method B and method C. A: Extraction solutions for this experiment were tested at dilutions of zero fold and one fold using method B. B: Extraction solutions were tested at dilutions of zero fold and one fold using method C.

Given that methods A and B (Figure 2A) failed to detect chlorpyrifos in *Folium Artemisiae Argyi*, a substitution with method C was used to elute impurities from Artemisia. The detection results for Artemisia are shown in Figure 2B. Figure 2B (dilutions of zero fold) shows false positive results for the control (CK) and spiked samples. This was corrected by diluting by a factor of one, as shown in Figure 2B (dilutions of one fold) where CK for *Folium Artemisiae Argyi* exhibits negative results while the spiked sample shows positive results, as described in Table 10. Therefore, method C was used for chlorpyrifos extraction from *Folium Artemisiae Argyi*, combined with a twofold dilution, effectively reducing the interference caused by *Folium Artemisiae Argyi* in the detection of chlorpyrifos via GICA.

**Table 10:** Detection results of chlorpyrifos using method C.

Name of Chinese medicinal herbs	Dilution of chlorpyrifos at 30 ng/ml in varying ratios			
*Folium Artemisiae Argyi*	1:0	CK	1:1	CK
Result	+	+	+	−

negative (−), positive (+), not detected (ND).

#### 3.2.2 Detection results of triazophos in four Chinese medicinal herbs

##### 3.2.2.1 Spiking test using method B

The results from method A extraction revealed false negatives in the spiking test strip results. Consequently, a switch to method B for extraction was implemented. *Polygonatum sibiricum*, *O. japonicus*, and *Bulbus Fritillaria* were spiked with concentrations of 100 and 200 ng/ml of Triazophos using method B. The results showed positive outcomes for all three medicinal herbs at both concentration levels, with the control samples of these three matrices yielding negative results. However, *Folium Artemisiae Argyi* spiked at 200 ng/ml and showed positive results. Upon reaching the detection limit of 100 ng/ml, false negatives were observed and all negative samples displayed true negative results, as detailed in Table 11. Similarly, employing method B for extraction showed that *Polygonatum sibiricum*, *O. japonicus*, and *Bulbus Fritillaria* had detection limits as low as 100 ng/ml, whereas *Folium Artemisiae Argyi*, due to matrix interference, had an increased detection limit of 200 ng/ml. To enhance the detection concentration of triazophos in *Folium Artemisiae Argyi*, method C was selected to test *Folium Artemisiae Argyi* spiked with a concentration of 100 ng/ml.

**Table 11:** Spiking test results using method B.

Name of Chinese medicinal herbs	The concentration of triazophos (ng/ml) compared to the CK		
	100	200	CK
*Polygonatum sibiricum*	+	+	−
*Ophiopogon japonicus*	+	+	−
*Bulbus Fritillaria*	+	+	−
*Folium Artemisiae Argyi*	−	+	−

negative (−), positive (+), not detected (ND).

##### 3.2.2.2 Spiking test using method C

Given the inability of method B to detect a concentration of 100 ng/ml in *Folium Artemisiae Argyi*, extraction was performed according to the guidelines of method C outlined in Section 2.3.3.3. Prior to dilution, both the blank and spiked samples yielded positive results. However, after dilution to a one fold level, the blank sample for *Folium Artemisiae Argyi* changed from positive to negative, while the spiked sample remained positive. Detailed results can be found in Table 12. These findings demonstrate the applicability of method C in extracting the triazophos pesticide at a concentration of 100 ng/ml in *Folium Artemisiae Argyi*.

**Table 12:** Results of triazophos with different dilution factorsin *Folium Artemisiae Argyi* using method C.

Name of Chinese medicinal herbs	Different dilution factors of triazophos at 100 ng/ml compared to the CK			
	CK		Spiking	
*Folium Artemisiae Argyi*	1:0	1:1	1:0	1:1
Results	+	−	+	+

#### 3.2.3 Detection results of isocarbophos in four Chinese medicinal herbs

##### 3.2.3.1 Selection of extraction method A for isocarbophos in four Chinese medicinal herbs

**Figure 3:**
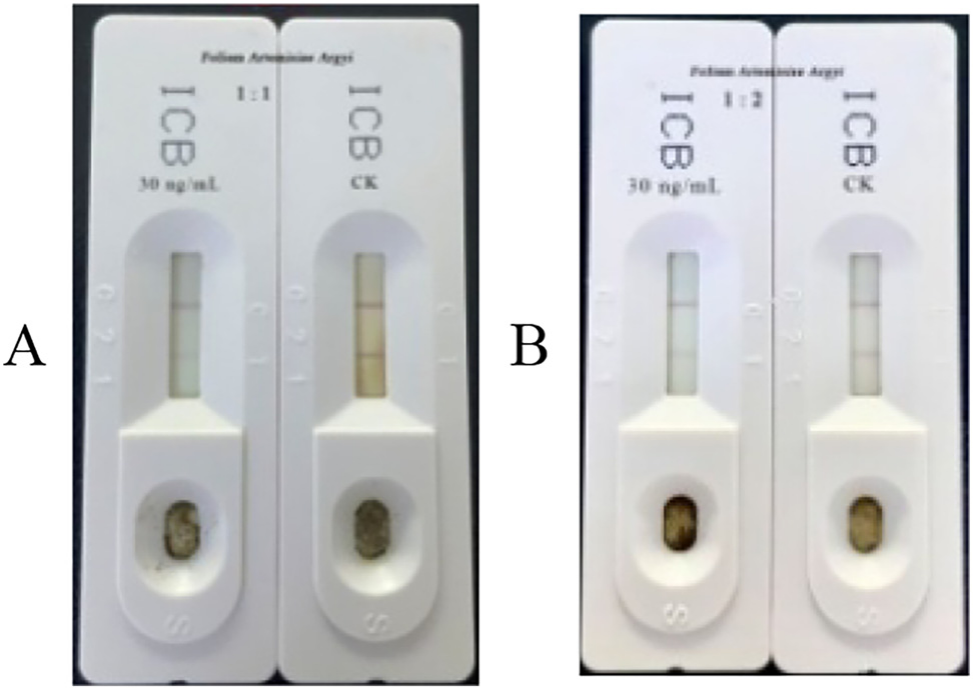
Detection results of 30 ng/ml isocarbophos at different dilution factors in *Folium Arte*misiae* Argyi*. A: Extraction solutions for this experiment were tested at dilutions of onefold. B: Extraction solution was tested at dilutions of twofold.

According to the control with standard for isocarbophos, adding 20–50 ng/ml of the isocarbophos standard solution to *Polygonatum sibiricum*, *O. japonicus*, *Bulbus Fritillaria*, and *Folium Artemisiae Argyi* produced the outcomes detailed in Table 13. When 30 and 50 ng/ml of isocarbophos were added to the matrices, the test strip exhibited positive results for all four matrices. However, when the concentration was decreased to 20 ng/ml, the detection results were negative. Therefore, 30 ng/ml represents the lowest detectable limit of isocarbophos in *Polygonatum sibiricum*, *O. japonicus*, and *Bulbus Fritillaria* (Table 13). *Folium Artemisiae Argyi* exhibited a false positive (CK), prompting a method involving dilution of the extraction solution. Dilution factors of 1, 2 were used (Figure 3A and B). All dilution factors showed negative CK results, with positive spiking results emerging with increasing dilution factor. When the dilution factor reached 1:5, the spiking test result turned negative. Thus, the optimal dilution factor chosen for the extraction solution of isocarbophos in *Folium Artemisiae Argyi* is 1:2 (Figure 3B). We offer a table summarizing which method works best for each herb-pesticide pair. (Table 14).

**Table 13:** GICA results of four Chinese herbal medicines spiking with different concentrations of isocarbophos using method A.

Name of Chinese medicinal herbs	The spiking concentration of isocarbophos (ng/ml) compared to the blank CK Unit (ng/ml)			
	50	30	20	CK
*Polygonatum sibiricum*	+	+	−	−
*Ophiopogon japonicus*	+	+	−	−
*Bulbus Fritillaria*	+	+	−	−
*Folium Artemisiae Argyi*	+	+	−	+

**Table 14:** Method works best for each herb-pesticide pair.

	*Polygonatum sibiricum*			*Ophiopogon japonicus*			*Bulbus Fritillaria*			*Folium Artemisiae Argyi*			Rationale
	Chlorpyrifos	Triazophos	Isocarbophos	Chlorpyrifos	Triazophos	Isocarbophos	Chlorpyrifos	Triazophos	Isocarbophos	Chlorpyrifos	Triazophos	Isocarbophos	
Method A	Y	ND	Y	Y	ND	Y	Y	ND	Y	ND	ND	Y	Method A is applicable to the extraction of chlorpyrifos and isocarbophos from some Chinese herbal medicines.
Method B	–	Y	–	–	Y	–	–	Y	–	ND	ND	–	Method B is applicable to the extraction of triazophos in three Chinese herbal medicines
Method C	–	–	–	–	–	–	–	–	–	Y	Y	–	Method C is applicable to the extraction of chlorpyrifos and triazophos from folium Artemisiae Argyi

detected (Y), not detected (ND), untried(−).

#### 3.2.4 Matrix effects of three pesticides in four different Chinese herbal medicinal matrices

Based on the colloidal gold test strip method, a study was conducted to investigate the MEs of three pesticide residues in four different Chinese herbal medicinal matrices. The analysis revealed three main types of MEs, as shown in Table 15. The first type exhibits a strong enhancement effect on the matrix. For example, when method A was used to extract the matrices of *Polygonatum sibiricum*, *O. japonicus*, and *Bulbus Fritillaria* with chlorpyrifos, it showed an enhancement effect (MEs < −50 %). The second type primarily demonstrates a moderate ME. For instance, when method A was used to extract the matrices of *Polygonatum sibiricum*, *O. japonicus*, *Bulbus Fritillaria*, and *Folium Artemisiae Argyi* with isocarbophos, it displayed a medium matrix-enhancement effect (−20 % > MEs > −50 %). The third type exhibits both weak and strong MEs depending on the matrix type. Using triazophos as an example, when method B was employed to extract the matrices of *Polygonatum sibiricum*, *O. japonicus*, and *Bulbus Fritillaria*, it showed negligible MEs (−20 % < MEs < 20 %); however, when extracting the *Folium Artemisiae Argyi* matrix, it displayed a strong matrix-inhibition effect (MEs > 50 %).

**Table 15:** Matrix effects of three pesticides in four different Chinese herbal medicinal matrices.

Extraction methods	Names of pesticide	Control with standard concentration (mg/ml)	*Polygonatum sibiricum*	*Ophiopogon japonicus*	*Bulbus Fritillaria*	*Folium Artemisiae Argyi*
Method A	Chlorpyrifos	250	−88 %	−88 %	−88 %	×
	Triazophos	100	×	×	×	×
	Isocarbophos	50	−40 %	−40 %	−40 %	−40 %
Method B	Chlorpyrifos	250	–	–	–	×
	Triazophos	100	0	0	0	100 %
	Isocarbophos	50	–	–	–	–
Method C	Chlorpyrifos	250	–	–	–	−88 %
	Triazophos	100				0
	Isocarbophos	50	–	–	–	–

×indicates that the method cannot extract and “–” indicates that the method was not used.

## 4 Discussion

This study seeks to improve the pretreatment method used for GICA to detect OP pesticide residues in Chinese medicinal herbs. The detection limit for chlorpyrifos in the standard control is 250 ng/ml. By adding 30 ng/ml of chlorpyrifos to the matrices of *Polygonatum sibiricum*, *O. japonicus*, and *Bulbus Fritillaria*, the pesticide could be accurately detected using method A, as outlined in the test strip standard practice instruction. However, for *Folium Artemisiae Argyi*, method C, which involves hexane: acetone (1:1) extraction followed by dilution, is required to eliminate MEs and achieve a detection limit of 30 ng/ml. The detection limit for triazophos in the standard control is 100 ng/ml. When extracting triazophos at a concentration of 100 ng/ml from the matrices of *Polygonatum sibiricum*, *O. japonicus*, and *Bulbus Fritillaria*, method B, which involves acetonitrile heating, is necessary. For extracting triazophos in *Folium Artemisiae Argyi*, the detection limit of 100 ng/ml is achieved through method C, which involves extraction and dilution by a factor of one. Isocarbophos has a detection limit of 50 ng/ml in the control standard. Method A can be used to extract isocarbophos from the matrices of *Polygonatum sibiricum*, *O. japonicus*, *Bulbus Fritillaria*, and *Folium Artemisiae Argyi*. The extraction liquids from the matrices of *Polygonatum sibiricum*, *O. japonicus*, and *Bulbus Fritillaria*can be directly tested on the test strip, resulting in a detection limit of 30 ng/ml.

However, the extraction liquids obtained from *Folium Artemisiae Argyi*should be diluted by a factor of two, resulting in a detection limit of 30 ng/ml. In the quantitative analysis of chlorpyrifos, triazophos, and isocarbophos, the extraction method fully eliminates matrix interference and increases the sensitivity of GICA compared with test strip standard practice instruction method was increased by approximately 8.3, 1.7, and 1 time, respectively. The composition of different Chinese herbal matrices varies, leading to different impacts on the three pesticides, including matrix-enhancement effects and interference effects. When *Folium Artemisiae Argyi* is used as the matrix, method A fails to extract chlorpyrifos and triazophos, exhibiting a strong inhibitory effect. Method B for triazophos extraction demonstrates a strong matrix-inhibition effect. Conversely, method C for chlorpyrifos and triazophos extraction shows a strong matrix-enhancement effect and a weak ME. This could be attributed to impurities in *Folium Artemisiae Argyi*, such as volatile oils, flavonoids, chlorogenic acid, and acidic polysaccharides. When *Polygonatum sibiricum*, *O. japonicus*, and *Bulbus Fritillaria* are utilized as matrices, method A for chlorpyrifos and isocarbophos extractions displays strong and moderate MEs, while method B for triazophos extraction exhibits a weak ME.

The quantitative assessment of trace pesticide residues in complex matrices, such as Chinese medicinal herbs, poses significant challenges due to their low water content and the presence of various unwanted components, including sugars, phenolic substances, flavonoids, natural pigments, and essential oils [26]. During the process of pesticide extraction, natural products and secondary metabolites present in Chinese medicinal herbs are co-extracted, and these co-extracts can have substantial interactions with immunoglobulins. Hence, it is reasonable to speculate that natural products and secondary metabolites may effectively bind with gold-labeled antibodies, resulting in MEs through epitope conformational distortions or blocking antigen–antibody reactions on the detection line. Furthermore, they may enhance antibody binding sites, facilitate antibody–antigen interactions, and consequently induce MEs. Methods to mitigate MEs involve improving sample preparation and diluting sample extracts [27], 28].

The MEs of Chinese medicinal herbs can affect the detection of pesticides using colloidal gold. This study aims to investigate pretreatment methods that can improve the accuracy of detection. The experiment compares the effects of three pesticide pretreatment methods on the levels of OP pesticide residues in Chinese herbal matrices.

(1). Method A extracts chlorpyrifos from the rhizomes and stems of Chinese medicinal herbs belonging to the Liliaceae family, such as *Polygonatum sibiricum*, *O. japonicus*, and *Bulbus Fritillaria.* It has a detection limit of 30 ng/ml and shows a matrix-enhancement effect. This effect could be attributed to specific components in the Liliaceae herbal matrices that increase the binding sites for antibodies, leading to enhanced adsorption of the target substances.
(2). Due to the MEs, method A is unable to extract triazophos residues from four matrices. However, method B can extract triazophos residues from matrices such as Liliaceae rhizomes and stems (*Polygonatum sibiricum*, *O. japonicus*, *Bulbus Fritillaria*), with a detection limit of 100 ng/ml. It is worth noting that method B demonstrates a significant ME.
(3). As the ME of *Folium Artemisiae Argyi*hinders the extraction of chlorpyrifos and triazophos, this study selects the method C (hexane: acetone) nitrogen blowing method for the extraction of chlorpyrifos and triazophos from *Folium Artemisiae Argyi.* The detection limits for chlorpyrifos and triazophos are determined to be 30 and 100 ng/ml, respectively. These results indicate a significant enhancement effect of the matrix and a minor inhibitory effect.

The ME of *Folium Artemisiae Argyi*was found to be particularly strong, as demonstrated by Zhang et al. [29], who identified chlorophyll as a key source of matrix interference in the immunoassay of cyanamide residues in vegetables. This experiment revealed that the high chlorophyll content in the Artemisia matrix resulted in a pronounced inhibition effect when detecting chlorpyrifos and triazophos using the colloidal gold method, consistent with the findings of Zhang et al. [29]. However, the detection of isocarbophos showed a moderate enhancement effect on the matrix, contradicting the results presented by Zhang et al. [29]. This discrepancy may be attributed to structural modifications in the antibodies induced by isocarbophos, underscoring the need for further investigation to validate this hypothesis.

Wu et al. [26] employed acidified acetonitrile as the extraction solvent in their study and observed a decrease in the efficiency of test strip performance. This decline can likely be attributed to the pH of the extraction solution, which affects the binding interactions between antigens and antibodies, as well as secondary antibodies. Consequently, the colloidal gold test strip detection method is deemed unsuitable for target extraction when acidified acetonitrile is employed. Method A is applicable to the extraction of chlorpyrifos and Isocarbophos from some Chinese herbal medicines. Method B is applicable to the extraction of triazophos in three Chinese herbal medicines. Method C is applicable to the extraction of chlorpyrifos and triazophos from complex matrices such as Folium Artemisiae Argyi. Next we plan to explain what causes matrix inhibition versus enhancement specifically in chemical composition data.

## References

[1] Bhatt P, Gangola S, Bhandari G, Zhang W, Chen S, Mishra S (2020). New insights into the degradation of synthetic pollutants in contaminated environments. Chemosphere.

[2] Mishra S, Zhang WP, Lin ZQ, Pang SM, Huang YH, Bhatt P (2020). Carbofuran toxicity and its microbial degradation in contaminated environments. Chemosphere.

[3] Zhan H, Huang H, Lin ZQ, Bhatt P, Chen SH (2020). New insights into the microbial degradation and catalytic mechanism of synthetic pyrethroids. Environ Res.

[4] Huang YH, Zhang WP, Pang SM, Chen JM, Bhatt P, Mishra S (2021). Insights into the microbial degradation and catalytic mechanisms of chlorpyrifos. Environ Res.

[5] Yang FW, Li YX, Ren FZ, Wang R, Pang GF (2019). Toxicity, residue, degradation and detection methods of the insecticide triazophos. Environ Chem Lett.

[6] Zhang H, Wang XQ, Zhuang SL, Jin N, Wang XY, Qian MR (2012). Enantioselective analysis and degradation studies of isocarbophos in soils by chiral liquid chromatography-tandem mass spectrometry. J Agric Food Chem.

[7] Geng LJ, Huang JC, Fang MG, Wang HF, Liu JJ, Wang GX (2024). Recent progress of the research of metal-organic frameworks-molecularly imprinted polymers (MOFs-MIPs) in food safety detection field. Food Chem.

[8] Cao YR, Feng TY, Xu J, Xue CH (2019). Recent advances of molecularly imprinted polymer-based sensors in the detection of food safety hazard factors. Biosens Bioelectron.

[9] Tong XY, Lin XF, Duan N, Wang ZP, Wu SJ (2022). Laser-printed paper-based microfluidic chip based on a multicolor fluorescence carbon dot biosensor for visual determination of multiantibiotics in aquatic products. ACS Sens.

[10] Geng LJ, Sun JH, Liu MY, Huang JC, Dong JW, Guo Z (2024). Molecularly imprinted polymers-aptamer electrochemical sensor based on dual recognition strategy for high sensitivity detection of chloramphenicol. Food Chem.

[11] Geng LJ, Wang HF, Liu MY, Huang JC, Wang GX, Guo Z (2024). Research progress on preparation methods and sensing applications of molecularly imprinted polymer-aptamer dual recognition elements. Sci Total Environ.

[12] Peltomaa R, Barderas R, Benito-Pena E, Moreno-Bondi MC (2022). Recombinant antibodies and their use for food immunoanalysis. Anal Bioanal Chem.

[13] Geng Z, Li XH, Gou Y, Gao BX, Qi JL, Zhong L (2020). Determination of 53 pesticide residues in different category of fritillaria by QuEChERS and GC-MS/MS. Chin Tradit Herb Drugs.

[14] Wang HY, Liu SY, Zhou MY, Zhang GY, Chen ZJ, Chen LF (2020). Determination of heavy metals and organochlorine pesticides in tetrastigma hemsleyanum from different place. Chin Herbal Medicines.

[15] Cui XW, Wang SY, Cao H, Guo H, Li YJ, Xu FX (2018). A review: the bioactivities and pharmacological, applications of *Polygonatum sibiricum* polysaccharides. Molecules.

[16] Yelithao K, Surayot U, Ju HL, You SG (2016). RAW264.7 cell activating glucomannans extracted from rhizome of polygonatum sibiricum. Prev Nutr Food Sci.

[17] Zhao XY, Li J (2015). Chemical constituents of the genus polygonatum and their role in medicinal treatment. Nat Prod Commun.

[18] Pharmacopoeia Commission of PRC (2020). Pharmacopoeia of the People’s Republic of China.

[19] Chen MH, Chen XJ, Wang M, Lin LG, Wang YT (2016). Ophiopogon japonicas-A phytochemical, ethnomedicinal and pharmacological review. J Ethnopharmacol.

[20] Zhu RW, Zheng CJ (2008). The incidence characteristics and prevention measures of pingbeimu rust disease. China Plant Prot.

[21] Pharmacopoeia Commission of PRC (2020). Pharmacopoeia of the People’s Republic of China.

[22] Geng LJ, Liu JJ, Zhang WB, Wang HF, Huang JC, Wang GX (2024). Preparation of dual recognition adsorbents based on molecularly imprinted polymers and aptamer for highly sensitive recognition and enrichment of ochratoxin A. J Hazard Mater.

[23] Wang XD, Lin H, Sui JX, Cao L (2013). The effect of fish matrix on the enzyme-linked immunosorbent assay of antibiotics. J Sci Food Agric.

[24] Yu F, Jia LJ, Huang W, Lin SY, Wang HB, Yang B (2014). Study the pretreatment method of the China traditional medicine in pesticide residue analysis. Agrochemicals.

[25] Li HX, Fan WF, Zheng YL, Wu MQ, Zhou MX, Chen XY (2022). Comparative analysis of 8 active ingredients in Artemisia argyi leaves from different habitats by HPLC. Lishizhen Med Mater Med Res.

[26] Wu JW, Xu R, Zhao RH (2011). Analysis of pesticide residues using the quick easy cheap effective rugged and safe (QuECh-ERS) pesticide multiresidue method in fifty traditional Chinese medicine by gas chromatography. J Anal Sci.

[27] Raposo F, Barcelo D (2021). Challenges and strategies of matrix effects using chromatography mass spectrometry: an overview from research versus regulatory viewpoints. TrAC, Trends Anal Chem.

[28] Rutkowska E, Łozowicka B, Kaczynski P (2019). Three approaches to minimize matrix effects in residue analysis of multiclass pesticides in dried complex matrices using gas chromatography tandem mass spectrometry. Food Chem.

[29] Zhang ZA, Lin H, Sui JX, Han XN, Wang LF, Sun X (2022). The effect of chlorophyll on the enzyme-linked immunosorbent assay (ELISA) of procymidone in vegetables and the way to overcome the matrix interference. J Sci Food Agric.

